# Oral versus intravenous clarithromycin in moderate to severe community-acquired pneumonia: an observational study

**DOI:** 10.1186/s41479-017-0025-2

**Published:** 2017-02-05

**Authors:** Nikolas Rae, Aran Singanayagam, Stuart Schembri, James D. Chalmers

**Affiliations:** 1 Scottish Centre for Respiratory Research, University of Dundee, DD1 9SY Dundee, UK; 2 Imperial College London, SW7 2AZ London, UK

**Keywords:** Macrolide, Pneumonia, Severity, Combination therapy, Antibiotic

## Abstract

**Objectives:**

British Thoracic Society guidelines recommend clarithromycin in addition to beta-lactam antibiotics for patients with community-acquired pneumonia and CURB-65 score 2–5. Intravenous therapy is commonly used but there are few data on whether oral therapy is equally effective.

**Methods:**

This observational study used propensity matching to compare two groups of patients with moderate to severe community-acquired pneumonia (CURB-65 score 2–5) treated with oral (*n* = 226) or intravenous (*n* = 226) clarithromycin on admission. Outcomes were 30-day mortality, intensive care unit admission, time to clinical stability, and length of hospital stay.

**Results:**

There was no significant difference in 30-day mortality (16.8% for intravenous [IV] group vs. 14.6% for oral group, hazard ratio for IV group 1.11 95% CI 0.70–1.78), ICU admission (10.6% in both groups) or complications (10.6% for IV group and 9.3% for oral group) between the groups. The time to clinical stability in both cohorts was a median of 5 days (interquartile range 3–7 days, *p* = 0.3). The median length of hospital stay was 8 days in the IV group (interquartile range 4–14 days) and 7 days in the oral group (interquartile range 4–13 days), *p* = 0.5. No other differences were observed between oral and IV groups.

**Conclusion:**

Where the oral route is not compromised, oral macrolides appear to be equivalent to IV in treating moderate to severe CAP.

## Introduction

Guidelines for treatment of community-acquired pneumonia (CAP) recommend adding an intravenous (IV) macrolide to a β-lactam agent (penicillin, penicillin/β-lactamase inhibitor combination or second/third generation cephalosporin) in the treatment of moderate to severe CAP [1, 2]. There is currently no evidence that the route of administration of macrolides alters clinical outcome.

Macrolides are commonly used in the management of respiratory tract infections, with particular activity against atypical organisms in addition to *Streptococcus pneumoniae*, *Haemophilus influenzae* and *Moraxella catarrhalis* [3, 4]. Oral clarithromycin has a bioavailability of approximately 55% and excellent pulmonary tissue penetration, achieving concentrations higher than those observed in plasma [5, 6]. Peak plasma concentrations are achieved within 2 h [6]. IV administration is more expensive and may be associated with a higher rate of adverse effects, thus administration by the oral route is preferable wherever possible [7, 8]. Recent randomized controlled trials have given conflicting data on the overall value of macrolides in the management of community-acquired pneumonia [9, 10]. A non-inferiority trial from Switzerland [9] comparing β-lactam monotherapy to β-lactam plus macrolide concluded that β-lactam monotherapy was not non-inferior in terms of time to clinical stability. A significant difference suggesting that macrolides improve rate of clinical recovery was demonstrated, driven by a higher effectiveness in a small group of patients with atypical pathogens [9]. In contrast, a cluster randomized controlled trial in The Netherlands [10] has recently reported no benefit in terms of mortality for hospitals randomized to a regime consisting of β-lactam plus macrolide, compared to β-lactams alone. None of these studies have addressed whether macrolides should be administered orally or intravenously. A recent study [11] has examined the administration of fluoroquinolones and concluded that administration of fluroquinolones orally was at least equivalent to IV administration in CAP patients.

The aim of this study was to ascertain if the route of administration of macrolides was associated with outcome in patients with moderate or severe community-acquired pneumonia.

## Methods

This study was a secondary analysis of a prospectively collected database (2005–2009) that has been described previously. Data collection was approved by the South East Scotland Research Ethics Committee (reference numbers S1104/15 and S1103/27).

## Inclusion and exclusion criteria

Patients were included in the study if they presented with new infiltrates on chest radiograph along with signs and symptoms suggestive of pneumonia [12]. In addition, patients were eligible for inclusion in the present analysis if they had a CURB-65 score of between 2 and 5 and received an IV β-lactam with clarithromycin (either IV or oral) as the initial antibiotic treatment regimen on admission, as per British Thoracic Society (BTS) guidelines [2].

Exclusion criteria were hospital-acquired pneumonia, active malignancy, immunosuppression, pulmonary embolism, tuberculosis, and patients in whom active treatment was not considered appropriate. In addition, for this analysis patients were excluded if the oral route was compromised or if they received a non-guideline concordant antibiotic regime.

## Propensity matching

The probability that a patient would receive oral or IV clarithromycin was assessed with multivariable logistic regression to create a propensity score [13]. The variables included in the propensity model were all of those available to clinicians at admission (symptoms, demographics, co-morbidities, clinical variables, laboratory results and radiology). Each patient treated with IV ß-lactam and oral clarithromycin was then matched to a patient treated with IV ß-lactam and IV clarithromycin with a similar propensity score, using greedy matching. This created two cohorts that were well matched for measured confounders. As a sensitivity analysis to exclude strong differential effects among patients that could not be matched, the analysis was also repeated including the propensity score as a covariate in the Cox proportional hazards regression [14].

The group who received initial IV β-lactam with IV clarithromycin are referred to in the manuscript as the IV group, and those who initially received IV β-lactam with oral clarithromycin are referred to as the oral group.

## Outcomes

The primary outcome was 30-day mortality, with secondary outcomes including length of hospital stay, intensive care unit (ICU) admission and development of empyema or complicated parapneumonic effusion.

## Statistical analyses

Statistical analyses were performed using SPSS version 21 (IBM, New York, United States). Propensity matching was performed using the propensity matching add-on for SPSS (SPSS essentials for R and R version 2.14.2). The propensity analysis is described above. Between the treatment groups, outcomes were assessed after multivariable adjustment using Cox proportional hazards regression. The multivariable analysis included age, gender and CURB-65 score. More extensive models including all variables in Table 1 were also tested to reduce unmeasured confounding. A sensitivity analysis was performed in patients with CURB-65 scores 3–5, as this group are recommended for IV macrolides in the BTS guidelines [2].

**Table 1:** Multivariable analysis of factors associated with intravenous macrolide treatment

Variable	Odds ratio (95% CI), *p*-value
Gender (male)	0.79 (0.55–1.13), *p* = 0.2
Age	1.01 (0.99–1.03), *p* = 0.1
Congestive cardiac failure	0.83 (0.51–1.35), *p* = 0.5
Liver disease	0.94 (0.44–2.00), *p* = 0.9
Stroke	1.23 (0.68–2.22), *p* = 0.5
COPD	0.80 (0.52–1.23), *p* = 0.3
Diabetes	1.06 (0.63–1.80), *p* = 0.8
Smoking status	1.04 (0.71–1.52), *p* = 0.8
CURB-65 score	1.13 (0.90–1.41), *p* = 0.3
Temperature	1.07 (0.89–1.29), *p* = 0.5
Pulse rate	1.0 (0.99–1.01), *p* = 0.4
Prior statin use	1.11 (0.71–1.75), *p* = 0.7
Antiplatelets	1.01 (0.65–1.56), *p* = 0.9
ACE inhibitors/ARBs	0.90 (0.56–1.45), *p* = 0.7
Beta-lactam co-administration	0.89 (0.56–1.41), *p* = 0.6
Corticosteroid use	0.90 (0.49–1.66), *p* = 0.7
Sodium level	0.99 (0.95–1.03), *p* = 0.5
Haemoglobin	1.00 (0.99–1.01), *p* = 0.4
White cell count	1.01 (0.99–1.02), *p* = 0.3
Platelet count	1.00 (0.99–1.00), *p* = 0.8
Albumin	1.0 (0.49–1.66), *p* = 0.7
Multilobar radiographic changes	1.14 (0.73–1.79), *p* = 0.6
Antibiotic treatment prior to admission	1.67 95% CI 0.96–2.92), *p* = 0.08

*COPD* chronic obstructive pulmonary disease, *ACE* inhibitors, angiotensin-converting-enzyme inhibitors, *ARBs*, angiotensin receptor blockers

## Results

### Patients

There were 1,113 patients who had a CURB-65 score 2–5 and were eligible for inclusion in the study. Of these, 761 patients received guideline concordant therapy consisting of a ß-lactam and a macrolide. There were 118 patients with a compromised oral route that were excluded (all had IV therapy). The final cohort for matching consisted of 226 patients in the oral clarithromycin group and 417 patients treated with IV clarithromycin. The dose administered was not recorded but local guidelines recommended 500mg twice daily for both oral and IV administration.

### Propensity matching

In the logistic regression analysis, IV therapy was not independently associated with any of the variables considered; the strongest relationship was with oral antibiotic therapy prior to admission (odds ratio for IV therapy, 1.67 95% CI 0.96–2.92) (Table 1).

There were no patients in the oral group that could not be matched and, therefore, 452 patients were included after propensity matching, with 226 patients in the IV group propensity matched to 226 patients in the oral group. There were no significant differences in demographics, or physiological or laboratory parameters between the two groups, as shown in Table 2.

**Table 2:** Characteristics of the IV and oral clarithromycin groups after propensity matching

Characteristics	IV macrolide	Oral macrolide	*p*-value
N	226	226	
Age	71 (59–78)	71 (62–79)	0.5
Gender	116 (51.3%)	121 (53.5%)	0.6
Confusion	40 (17.7%)	41 (18.1%)	0.9
Respiratory rate	30 (20–32)	30 (20–33)	0.7
SBP	108 (91–129)	108 (90–130)	0.9
Temperature	38 (37–38.4)	38 (37–38.4)	0.9
Pulse	105 (90–120)	105 (90–120)	0.7
H+	38 (34–43.7)	38 (34–43)	0.4
Urea	8.2 (6.4–11.7)	8.5 (6.7–11.9)	0.4
Sodium	136 (133–138)	136 (133–138)	0.8
Glucose	6.6 (5.7–8.0)	6.9 (5.7–8.5)	0.2
CRP	231 (102–339)	209 (94–352)	0.7
WCC	15.3 (11.5–20.0)	15.1 (10.1–19.4)	0.6
Albumin	36 (32–39)	36 (33–40)	0.5
CURB-65	3 (2–3)	3 (2–3)	0.1

Data are median IQR except gender, which is presented as n (%)

*SBP* systolic blood pressure, *CRP* C-reactive protein, *WCC*, white cell count, *IV* intravenous, *IQR* interquartile range

### Outcomes: propensity matched cohort

There were 38 deaths (16.8%) in the IV group and 33 deaths (14.6%) in the oral group (*p* = 0.5). There was no significant difference in 30-day mortality of multivariable analysis (hazard ratio [HR] for IV group 1.11 95% CI 0.70–1.78). The time to clinical stability was a median of 5 days in both cohorts (interquartile range [IQR] 3–7 days, *p* = 0.3).

**Fig. 1:**
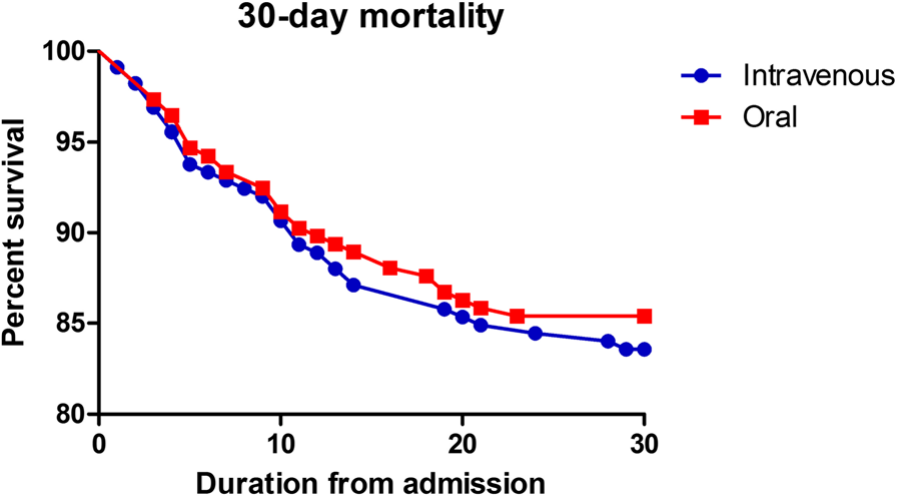
Kaplan-Meier plot of 30-day mortality between patients receiving oral or intravenous clarithromycin for moderate to severe community-acquired pneumonia

There were 24 patients (10.6%) who were admitted to the ICU >24 h after admission in both groups (*p* = 1.0) with no differences between the groups evident in multivariable analysis (HR 1.07 95% CI 0.72–1.59). There were 24 patients (10.6%) who developed empyema or complicated parapneumonic effusion in the IV group compared to 21 (9.3%) in the oral group (*p* = 0.6) with no difference between the groups on multivariable analysis (HR 1.06 95% CI 0.59–1.91). The median length of hospital stay was 8 days in the IV group (IQR 4–14 days) and 7 days in the oral group (IQR 4–13 days), *p* = 0.5. Figure 1 shows a Kaplan-Meier curve of 30-day mortality for the two groups.

In the analysis limited to patients with CURB-65 scores 3–5 (*n* = 272), the hazard ratio for mortality was 1.16 (0.67–2.03) and for ICU admission was 1.14 (0.73–1.78).

### Outcomes: sensitivity analysis

To exclude strong confounding effects from patients excluded from the propensity analysis, we performed a Cox proportional hazard regression including all patients (*n* = 643) considered for inclusion in the propensity analysis. In this analysis, the hazard ratios were similar to the primary analysis: 30-day mortality HR for IV therapy was 1.14 (95% CI 0.74–1.76); 30-day mortality HR for ICU admission was 1.16 (95% CI 0.83–1.62) and 30-day mortality HR for complicated pneumonia was 1.18 (95% 0.71–1.98), compared to oral therapy.

## Discussion

The addition of an IV macrolide to a β-lactam agent in the treatment of moderate or severe CAP is recommended in national and international guidelines [1, 2]. This study indicates that in patients with moderate or severe CAP where the oral route is not compromised, treatment with oral clarithromycin is as effective as IV clarithromycin when combined with an IV β-lactam. Oral therapy has several theoretical advantages, including reducing costs and risk of complications of IV administration of drugs [15].

Macrolides are primarily added to provide cover for atypical pathogens. Atypical pathogens are not associated with bacteraemia, and bacteraemia is the only circumstance where prompt IV antibiotic therapy is shown to give a mortality benefit [16]. Clarithromycin has a high bioavailability and achieves a peak serum concentration 2 h following oral administration [5]. The highly lipophilic nature of macrolides result in excellent tissue penetration irrespective of route of administration, with clarithromycin achieving concentrations up to 20 times greater in pulmonary epithelial tissues than in serum. Since IV clarithromycin is typically administered over 1 h, the difference in time to reach peak serum concentrations is highly unlikely to be clinically relevant.

In the UK, IV clarithromycin is at least 10 times more expensive than oral clarithromycin [17]. This cost does not include the time required for preparation of IV medications or consumables used for IV administration of drugs. CAP is common and even relatively small increases in the use of oral clarithromycin would result in significant cost savings.

There has been an ongoing debate about the relative value of macrolides in the management of CAP [9, 10]. Although some observational studies suggest a mortality benefit with macrolide containing regimes, a meta-analysis of observational studies could not conclude a definite benefit associated with macrolides in CAP [18] and a Cochrane review of randomized controlled trials (primarily of fluroquinolones) have failed to demonstrate a benefit for giving empirical atypical coverage in CAP [19]. Macrolides are now known to be associated with significant adverse effects including the induction of antibiotic resistance and *Clostridium difficile* [20–23]. Macrolides have been linked with cardiovascular events, although this association is controversial [24, 25]. While attention has focused on the potential for macrolides to prolong the QT interval or destabilize atherosclerotic plaques, a more simple explanation for some of the reported events is the large volume IV infusions required to administer. The question of the clinical value of macrolides will not be resolved without definitive randomized controlled trials. Two randomized trials have recently evaluated this question but have given somewhat conflicting results. Garin et al [9] could not demonstrate non-inferiority of ß-lactam alone compared to combination therapy with a primary outcome of time to clinical stability. This is not the same as saying that macrolide therapy speeds up clinical recovery, but this was certainly the case for a subgroup of patients with atypical infection in this study [9]. Larger studies would be needed to evaluate if macrolide indeed result in more rapid clinical response. Postma et al [10] reported a cluster randomized trial in The Netherlands where hospitals were randomized to a strategy consisting of ß-lactam plus macrolide, ß-lactam alone or fluoroquinolone. The study found that hospitals had similar mortality rates regardless of the strategy used, with no mortality benefit for macrolides demonstrated [10]. Limitations of the study included the cluster design that allowed significant deviations from the assigned antibiotic regime and a population of patients with predominantly mild disease.

Therefore, while awaiting a definitive answer to the question of which patients benefit from macrolide therapy, it is possible to minimise the harm associated with macrolides by following guideline recommendations for these agents, using the shortest duration necessary and, as this study shows, some harms and costs may be minimized by using oral therapy in preference to IV administration.

Limitations of this study must be acknowledged. This is an observational study, and although the oral and IV groups were well matched after excluding patients too severely ill to take oral medications, unmeasured confounding may remain. A randomized controlled trial would be required for confirmation. This study is the largest to address this question, but would be underpowered to detect small differences in outcome between the groups. Sample size was determined based on the available data. Nevertheless, it must be noted that none of the hazard ratios suggested a harmful effect of oral therapy.

## Conclusion

This observational study suggests that route of administration of clarithromycin in patients with moderate or severe CAP is not associated with a difference in clinical outcome, in patients able to take oral therapy. This would justify a definitive randomized controlled trial.

## Abbreviations

CAP: Community-acquired pneumonia
CRP: C-reactive protein
CURB-65: Confusion, urea, respiratory rate, blood pressure, age > 65
HR: Hazard ratio
ICU: Intensive care unit
IQR: Interquartile range
IV: Intravenous
SBP: Systolic blood pressure
WCC: White blood cell count
